# Single-nucleus RNA sequencing of midbrain blood-brain barrier cells in schizophrenia reveals subtle transcriptional changes with overall preservation of cellular proportions and phenotypes

**DOI:** 10.1038/s41380-022-01796-0

**Published:** 2022-10-03

**Authors:** Sofía Puvogel, Astrid Alsema, Laura Kracht, Maree J. Webster, Cynthia Shannon Weickert, Iris E. C. Sommer, Bart J. L. Eggen

**Affiliations:** 1 Department of Biomedical Sciences of Cells and Systems, section Cognitive Neuroscience, University of Groningen, University Medical Center Groningen, Groningen, The Netherlands; 2 Department of Biomedical Sciences of Cells and Systems, section Molecular Neurobiology, University of Groningen, University Medical Center Groningen, Groningen, The Netherlands; 3 Laboratory of Brain Research, Stanley Medical Research Institute, Rockville, MD USA; 4 Schizophrenia Research Laboratory, Neuroscience Research Australia, Sydney, NSW Australia; 5 School of Psychiatry, University of New South Wales, Sydney, NSW Australia; 6 Department of Neuroscience and Physiology, Upstate Medical University, Syracuse, NY USA; 7 Department of Psychiatry, Brain Center Rudolf Magnus, University Medical Centre Utrecht, Utrecht, The Netherlands

**Keywords:** Schizophrenia, Cell biology, Molecular biology, Neuroscience

## Abstract

The midbrain is an extensively studied brain region in schizophrenia, in view of its reported dopamine pathophysiology and neuroimmune changes associated with this disease. Besides the dopaminergic system, the midbrain contains other cell types that may be involved in schizophrenia pathophysiology. The neurovascular hypothesis of schizophrenia postulates that both the neurovasculature structure and the functioning of the blood-brain barrier (BBB) are compromised in schizophrenia. In the present study, potential alteration in the BBB of patients with schizophrenia was investigated by single-nucleus RNA sequencing of *post-mortem* midbrain tissue (15 schizophrenia cases and 14 matched controls). We did not identify changes in the relative abundance of the major BBB cell types, nor in the sub-populations, associated with schizophrenia. However, we identified 14 differentially expressed genes in the cells of the BBB in schizophrenia as compared to controls, including genes that have previously been related to schizophrenia, such as *FOXP2* and *PDE4D*. These transcriptional changes were limited to the ependymal cells and pericytes, suggesting that the cells of the BBB are not broadly affected in schizophrenia.

## Introduction

Schizophrenia is a heterogeneous and often severe mental disorder, with a neurodevelopmental origin and an underlying polygenic architecture (reviewed in [1]). Psychotic symptoms are recurrent in patients with schizophrenia, and are associated with hyper-activity and hyper-reactivity of the midbrain dopaminergic neurons (reviewed in [2]). In addition, increased expression of pro-inflammatory cytokines, such as SERPINE3, IL1B and IL6, was observed in the midbrain of patients with schizophrenia [3–5]. Increased chronic inflammation may have detrimental consequences on midbrain vasculature, either during the increased inflammatory state, or once inflammation has subsided [6]. On the other hand, intrinsic alterations in the brain vasculature of patients with schizophrenia may impair toxin efflux and favor the ingression of toxic material into the central nervous system (CNS), leading to neuroinflammation (discussed in [7–9]). Also, alterations in the brainstem blood perfusion, which may be modulated by the brainstem vasculature, were described in schizophrenia and people at ultra-high risk for developing psychosis [10, 11]. Thus, the midbrain constitutes an area of interest for the study of schizophrenia and may exhibit alterations in its vasculature associated with the disease. Transcriptomic changes in the midbrain vasculature of schizophrenia patients may help identify possible mechanisms that contribute to schizophrenia brain pathophysiology.

Because of its high energy and oxygen demands, the brain is highly vascularized [12]. The blood vessels of the brain are arranged in the neurovascular unit (NVU), comprised of endothelial cells, contracting cells that regulate local brain blood flow such as smooth muscle cells and pericytes [13], fibroblasts, microglia, astrocytes, and neurons [14]. Astrocytes display great morphological and functional diversity (reviewed in [15]), which has led to their classification into different sub-populations. Protoplasmic astrocytes are in the gray matter of the brain and in close contact with neurons. Their end-feet wrap the brain vasculature [16] and contribute to the regulation of regional blood flow in response to changes in neuronal activity [17]. Fibrous astrocytes are in the brain white matter and express the glial fibrillary acid protein (GFAP) at higher levels as compared to protoplasmic astrocytes [18]. In response to disease or injury, astrocytes undergo morphological, molecular, and functional remodelling, including higher proliferation rates, production of cytokines and recruitment of immune cells [19] (discussed in [20]).

During CNS development (~15 g.w in humans [21, 22]), the cells of the NVU induce and modulate the expression of adherent and tight junction proteins in the brain endothelium, forming a blood-brain barrier (BBB) that provides a dynamic interface between the CNS and the periphery [23], and restricts the migration of cells and molecules into the brain [24]. *Post-mortem* inspections of brain tissue indicated a reduced expression of the tight junction protein Claudin-5 in hippocampal blood vessels of patients with schizophrenia [25], suggesting a possible disruption of hippocampal BBB integrity. Evidence derived from blood and cerebrospinal fluid (CSF) measurements indicated increased levels of adhesion molecules, such as sP-selectin and sICAM, in schizophrenia [26–28], which may favor the ingression of immune cells into the brain [29–31], and higher levels of albumin in CSF as compared to controls, suggesting increased BBB permeability in schizophrenia patients [26, 27, 32]. A higher density of CD163^+^ macrophages was reported in the subependymal zone of patients with schizophrenia [33], suggesting increased ingression of immune cells into the brain parenchyma and supporting the hypothesis of a less stringent BBB function in schizophrenia. In addition, a recent study using dynamic contrast-enhanced magnetic resonance imaging provided in-vivo evidence for increased BBB permeability in the thalamus of patients with schizophrenia [34].

To identify the altered molecular processes underlying the putative increased BBB permeability observed in schizophrenia, particularly in the brain vasculature, Harris et al. [35] performed RNA sequencing of micro dissected cortical brain vessels and identified transcriptional changes related to inflammatory processes in the vessels of patients with schizophrenia [35]. Nonetheless, no change reached significance when correcting for multiple comparisons, suggesting that potential alterations in the brain vasculature might be weak or inconsistent across the patients. Conversely, immunohistochemical staining and in situ hybridization showed increased expression of inflammation-related genes associated with schizophrenia, such as *HP, S100A9*, *CD163*, and *IFITM*, in hippocampal and cortical blood vessels [36, 37]. However, the proper functioning of the BBB involves a variety of cell types, and it is not known which cell type(s) of the BBB are particularly affected in schizophrenia.

Single cell or nucleus RNA sequencing (sc/snRNAseq) enables transcriptional profiling of different cell types at single-cell resolution [38]. scRNAseq provides information about the transcriptional heterogeneity of cells and tissues, and allows for the identification of cellular sub-populations that are associated with disease [39], developmental stages [40] or brain region [41]. Single-nucleus RNA sequencing (snRNAseq) is a powerful strategy to generate single cell transcriptomes from archived and well-characterized frozen tissues [42–44]. To our knowledge, two articles using snRNAseq of *post-mortem* samples of schizophrenia cases have been published [45, 46]. These studies profiled nuclei isolated from cortical tissue without enrichment strategies for cell type-specific nuclei, possibly precluding the detection of transcriptional alterations in less abundant cell populations like the cells of the BBB.

In the present study, we combined a fluorescence activated sorting isolation strategy with snRNAseq to characterize the cells of the BBB in the midbrain of schizophrenia (*n* = 15) and matched controls (*n* = 14). We analyzed the nuclei of ependymal, pericytes, SMCs, fibroblasts, astrocytes, and endothelial cells. We obtained a large number of nuclei, allowing the identification of different cellular sub-populations of the BBB, including different endothelial and astrocyte sub-populations. These data provide a fine-grained cellular and molecular characterization of the human midbrain and serve as a starting point to investigate the status and heterogeneity of the different BBB cell types in schizophrenia.

## Materials and methods

### Human brain tissue

**Fig. 1:**
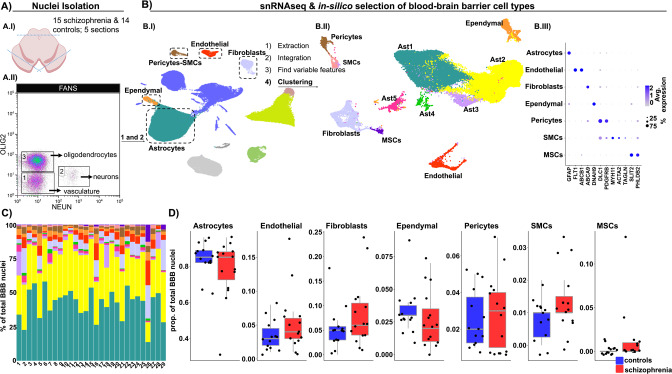
Identification of the main BBB cell types and their proportions in schizophrenia and controls. **A** Experimental workflow. **A.I** Representative illustration of the midbrain sections. Blue lines indicate the trimmed regions from the sections. **A.II** Fluorescence Activated Nucleus Sorting (FANS) isolation strategy to enrich for BBB nuclei. Double negative nuclei (DN), not expressing OLIG2 nor NEUN (gate 1), and NEUN positive nuclei (gate 2) were collected and snRNA sequenced in a ratio of ~6 DN to 1 NEUN nucleus. **B** **B.I** UMAP depicting 178,009 nuclei from 29 subjects (15 schizophrenia and 14 controls). Subsequent analyses were carried out considering only the BBB cell types (highlighted inside black dotted rectangles). BBB cell types were annotated according to the expression of cellular-specific genetic markers and extracted from the complete data set (in-silico cell types selection). **B.II** UMAP depicting 57,522 BBB nuclei. Colors indicate nuclei clusters resulting from unsupervised clustering analysis of nuclei transcriptomic profiles (dim = 50, k = 20, res = 0.1). **B.III** Dotplot depicting representative marker genes of each of the identified BBB cell types; the five sub-clusters of astrocytes in (**B.II**) were combined into one cluster. Dot size indicates the fraction of nuclei expressing the gene and the color depicts the gene scaled average expression. **C** Barplot depicting the percentages of the different BBB cell types per donor (*x* axis). **D** Boxplots depicting cell type proportions in schizophrenia and controls. Each dot indicates a sample and horizontal lines indicate the median. The five sub-clusters of astrocytes (**B.II**) were combined into one cluster. Group comparison was carried out with a generalized linear model and *p* values were Bonferroni corrected. No significant (adjusted *p* value < 0.05) differences were observed between schizophrenia and controls.

Midbrain samples from 15 schizophrenia and 14 control cases were obtained from the Stanley Medical Research Institute (SMRI) Array Collection (S. Table 1). The sample size was defined based on previous snRNAseq studies of human *post-mortem* brain tissue [46–48], and the cases were arbitrarily selected from the 35 schizophrenia and 35 controls of the SMRI Array Collection. *Post-mortem* brains were obtained from Medical Examiners with the permission from the next-of-kin. At least two senior psychiatrists independently made a psychiatric diagnosis (DSM IV) after review of all medical records and interviews with the family members. Ethical approval for the brain collection was through the Uniformed Services University for Health Sciences. Five 100 μm sections were used from each frozen midbrain block. The peduncles and colliculi were removed, retaining the ventral tegmental area and the substantia nigra (Fig. 1A.I). To ensure the quality of the brain tissue, RNA was isolated from the trimmed sections adjacent to the sampled tissue, using RNeasy Lipid Tissue mini kit (Qiagen, 74804) and RNA concentration and integrity were measured on a Bioanalyzer 2100 (Aligent). The average RIN value of the included samples was 7.42 ± 1.19, and all of them presented a RIN value > 4 (S. Table 1).

### Nuclei isolation

Nuclei were isolated from five midbrain sections per case as described in [49]. After sucrose density centrifugation, nuclei were incubated with fluorescently-conjugated antibodies directed against neuronal marker NEUN (RBFOX3/NEUN 1B7 AF647 mouse mAB, Novus Biologicals, NBP1-92693AF647) and the transcription factor OLIG2 for the oligodendrocyte lineage (Anti-OLIG2 clone 211F1.1 AF488 mouse mAB, Merck Millipore, MABN50A4). For each sample, we sorted DAPI^pos^NEUN^neg^OLIG2^neg^ (double negative nuclei; enriched for BBB cell types) and DAPI^pos^NEUN^pos^OLIG2^neg^ nuclei (neuronal nuclei) for single snRNAseq. The ratio of sorted and sequenced double negative to neuronal nuclei was set to ~6–1 (~39,300–6719 nuclei, S. Table 1).

### snRNAseq library construction and sequencing

Single-nucleus cDNA libraries were constructed according to the user guide of Chromium Single Cell 3′ Reagents Kit v3 (10× Genomics). All samples were pooled in equimolar ratios and sequenced on a NextSeq 500 at GenomeScan B.V. in Leiden and the Research Sequencing Facility of the UMCG, Groningen, The Netherlands. The median sequencing depth was 210 million reads per sample, and the median counts per nuclei was 2714. Authors were blinded during sample processing, 10× runs and library preparation.

### snRNAseq data analysis

Sequencing reads were processed and aligned to the GRCh38 human genome using Cell Ranger 3.0.1 [50] together with the pre-mRNA package to include both exonic and intronic reads. Barcode filtering was performed with Abacus [51], to distinguish barcodes containing nuclear RNA from cytoplasmic and ambient RNA. The following thresholds were set to keep high quality nuclei and remove cellular debris: (1) >100 exonic reads; (2) >200 intronic reads; (3) intronic reads > exonic reads. The counts corresponding to the barcodes passing the quality filters were extracted from the raw count matrix generated by Cell Ranger and loaded in R with Seurat v4.0. Nuclei with mitochondrial content >5% were removed. Count information from the 29 cases was log normalized using Seurat. Integration of the normalized data derived from the different cases was performed according to guidelines for fast integration with reciprocal PCA (rPCA) in Seurat, and Scrublet v0.2.1 was used to remove doublets. One small cluster of nuclei expressing both astrocytic and microglia marker genes was manually excluded due to the high chance of containing doublets. After these pre-processing steps, the mean number of reads per nuclei was 4802. Unbiased clustering analysis followed by the examination of expression of marker genes was used to identify all the major brain cell types.

### Selection of blood-brain barrier nuclei

From the complete snRNAseq dataset we extracted the clusters containing BBB cell types (Fig. 1B.I; endothelial, pericytes-SMCs, astrocytes, ependymal, and fibroblasts; a total of 71,766 nuclei). As for the complete dataset, counts from the 29 cases were log normalized and integrated with rPCA. Unbiased clustering analysis was performed with the Seurat workflow (clustering resolutions are indicated in the corresponding figures legends), and expression of marker genes was used to identify all the major BBB cell types (Fig. 1B.II–B.III). A cluster of 14,244 low quality nuclei with reduced average number of counts and features regarding the other clusters (<1050 and <850, respectively), was excluded for downstream analysis.

### Sub-clustering

We performed sub-clustering analysis only in the major BBB clusters containing >1000 nuclei. For sub-clustering of astrocytes, the nuclei of interest were extracted from the Seurat object and the counts from the 29 cases were re-integrated using canonical correlation analysis. Next, nuclei were sub-clustered with the default Seurat workflow. Two small sub-clusters of doublets (109 and 711 nuclei) were removed because of the expression of microglia and neuronal marker genes, respectively. For sub-clustering analysis on the other BBB cell types (endothelial, pericytes, fibroblasts, and ependymal nuclei), separate Seurat objects were made per cell type. The sub-clustering was performed considering the highly variable genes per cell type, now without re-integration step because there was no main effect of the cases.

### Quantification and statistical analysis

#### Demographics

To compare the mean of each case-related quantitative variable (S. Table 1) between the two diagnoses (schizophrenia and control), a parametric (Student’s *t* test) or a non-parametric test (Mann–Whitney U test) was used, depending on the data distribution. We used a Fisher’s exact test to evaluate the dependency between sex and diagnosis. The two groups presented equal variance and were not statistically different in any of the case-related variables (S. Table 2).

#### Marker genes identification

The function *FindAllMarkers* from Seurat v4.0 with default parameters was used to identify only positive differentially expressed genes (DEGs) per cluster (marker genes). *p* values were adjusted for multiple comparisons with the Bonferroni method. Marker genes per cluster are provided in S. Table 3 (marker genes of all BBB cell types), S. Table 4 (marker genes of endothelial sub-populations), and S. Table 5 (marker genes of astrocyte sub-populations).

#### Module scores

Gene sets module scoring was performed with the *AddModuleScore* function of Seurat, using default parameters.

#### Identification of differentially expressed genes between schizophrenia and control samples across the major blood-brain barrier cell types

To test for differences in the transcriptomic profiles between schizophrenia and controls, we made a prior selection of genes per BBB cell type that met the following conditions: (1) expressed at least in 25% of the nuclei in one of the two groups (schizophrenia or control); (2) With absolute log_2_FC > 0.3 between the two groups. Only expression data derived from samples that contributed with more than three nuclei to the given cell type were considered. The *zlm* function of the R package MAST v1.16.0 [52] was used to identify DEGs between schizophrenia and controls, across the different major BBB cell types. According to the MAST guidelines, we corrected gene expression by the cellular detection rate. We used a mixed linear model to account for donor-related structure in the data by including a random intercept per case. The results of this analysis are provided in S. Table 6. Genes were considered differentially expressed when the effect of diagnosis had a log2 fold change > 0.25, with a false discovery rate adjusted *p* value < 0.05. To evaluate if the expression of the identified DEGs was related to other case-related variables than diagnosis, we calculated the Pearson or Spearman correlation between the average expression of the DEG in the sample and every case-related variable. For sex, a categorical case-related variable, a point-biserial correlation was performed. Results of this correlation analysis are provided in S. Table 7.

#### Gene ontology enrichment analysis

Gene ontology (GO) enrichment analysis was performed on the abundantly expressed genes of each sub-population, using the *gost* function of the R package gprofiler2 v0.2.1, and *p* values were adjusted for multiple comparisons using correction_method = g_SCS. Because endothelial sub-clusters presented a great number of marker genes, we only used marker genes with log_2_FC > 1 and adjusted *p* value < 0.05, in order to facilitate sub-population identification. For GO analysis across astrocyte sub-populations, we used marker genes with log_2_FC > 0.5 and adjusted *p* value < 0.05. Significantly enriched GO terms per endothelial and astrocyte sub-clusters are provided in S. Table 8 and S. Table 9, respectively. Redundancy of enriched biological processes GO terms was accounted for with clustering analysis and aggregating terms with high semantic similarity, using the functions *calculateSimMatrix*, setting ont = “BP”, and *reduceSimMatrix* with threshold = 0.7 of the rrvgo v1.2.0 R package.

#### Comparisons of clusters and sub-cluster proportions between schizophrenia and controls

A generalized linear model (GLM) was used to test if the diagnosis (schizophrenia or control) affects the probability of a nucleus belonging to a given cluster. The GLM approach was previously used to estimate effects of diagnosis and batch on scRNAseq cluster proportions [53]. We used the *glmer* function of the lme4 R package 1.1.27.1 in R with a quasibinomial distribution, because of the binary nature of the response variable (the nucleus either belongs to the given cluster or not). Considering that a model was created per cluster, the obtained *p* values were corrected with the Bonferroni method, and the number of comparisons was set to the total number of clusters (or sub-clusters).

## Results

### Single-nucleus RNA sequencing and identification of major brain cell types

To sequence a relatively higher proportion of the cell types comprising the BBB, fluorescence activated nuclear sorting was combined with snRNAseq [49] in *post-mortem* midbrain sections of 15 schizophrenia and 14 control cases (Fig. 1A.I–A.II). Unbiased cluster analysis of nuclear transcriptomic profiles from 178,009 single nuclei revealed 19 clusters (S. Fig. 1A). These were annotated into 11 different main brain cell types based on the expression of cell type-specific marker genes (S. Fig. 1B). The majority of the sequenced nuclei derived from astrocytes (36.3% ± 14.6, S. Table 10). A cluster containing both pericytes and SMCs nuclei was identified based on the co-expression of *PDGFRB* and *ACTA2* (S. Fig. 1B). Fibroblasts and endothelial nuclei highly expressed *LAMA2* and *PECAM1*, respectively. Ependymal nuclei were identified because of their high expression of Doublecortin Domain-Containing Protein 1 (*DCDC1*) (S. Fig. 1B), in addition to their enrichment in genes expressed by mouse ependymal cells (S. Fig. 1C) [53].

### Transcriptional changes associated with schizophrenia are limited and specific to the ependymal cells and pericytes

To evaluate potential contribution of the cells of the BBB to schizophrenia brain pathology, we first extracted pericytes-SMCs, fibroblasts, astrocytes, ependymal, and endothelial nuclei (57,522 nuclei in total) from the complete dataset and determined variable genes within this selection, in order to identify different cellular populations (Fig. 1B.I, B.II). Unsupervised clustering of the BBB nuclei resulted in 11 clusters that were annotated into 7 main BBB cell types (Fig. 1B.III). The proportions of the different BBB cell types are indicated in S. Table 11. Pericytes (2.7% ± 1.7) and SMCs (1.1% ± 0.8) nuclei segregated into two different clusters; pericytes highly expressed *DLC1* and *PDGFRB*, whereas SMCs highly expressed *MYH11*, *ACTA2*, and *TAGLN* (Fig. 1B.III, S. Table 3). Ependymal nuclei (3% ± 2), probably derived from the cerebral aqueduct, highly expressed the cilia-related gene *DNAH9*. Fibroblasts (6.9 ± 5.9) abundantly expressed *ABCA9*, and endothelial nuclei (4.4 ± 3.6) were identified based on the expression of endothelial marker genes, such as *FLT1* [54]. We identified a small cluster of mesenchymal nuclei (MSCs; 0.79 ± 2.48) expressing *SLIT2* and *PHLDB2* (Fig. 1B.III, S. Table 3). Astrocytes comprised 81.1% (±13.5) of the BBB nuclei and segregated into five different clusters (Fig. 1B.II–BIII). No prominent donor effects were observed, as all samples contributed to nearly all clusters (Fig. 1C).

**Fig. 2:**
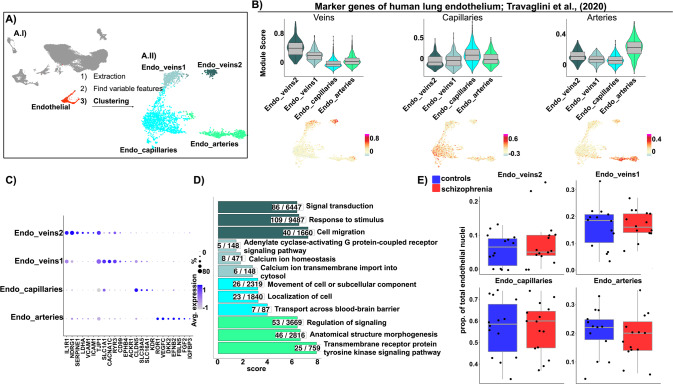
Identification and characterization of four endothelial sub-populations. **A** Sub-clustering workflow. **A.I** UMAP highlighting 2244 endothelial nuclei in red, which were extracted and used for sub-clustering analysis. **A.II** UMAP depicting four endothelial sub-clusters (dim = 50, k = 20, res = 0.1). **B** Top: Violin with boxplots depicting module scores for gene sets associated with human lung endothelial sub-populations, Travaglini et al., (2020), in human midbrain endothelial sub-clusters. Bottom: UMAP plots depicting the module score in each nucleus. **C** Dotplot depicting scaled average expression of representative marker genes of the identified endothelial sub-populations. **D** Barplot depicting the top three most significantly enriched gene ontology terms, grouped by biological processes, for the more abundantly expressed genes in each endothelial sub-population. Redundancy of the terms was reduced by aggregating terms with high semantic similarity. Score: negative logarithm_10_ of the adjusted *p* value resulting from the enrichment analysis. **E** Boxplots depicting endothelial sub-population proportions in schizophrenia and controls. Each dot indicates a sample and horizontal lines indicate the median. Group comparison was performed with a generalized linear model and *p* values were Bonferroni corrected. No significant (adjusted *p* value < 0.05) differences were observed between schizophrenia and controls.

To test for differences in the relative abundance of the major BBB cell types between schizophrenia and controls, we used a GLM, and no significant differences were observed (Fig. 1D). Next, we compared the transcriptomic profiles of the BBB cells in schizophrenia with respect to controls. Differences in the transcriptome between schizophrenia and controls were evaluated in each of the cell types, independently. With an absolute log_2_FC > 0.3 and adjusted *p* value < 0.05, we identified 14 differentially expressed genes (DEGs; S. Fig. 2A–D). The largest difference in gene expression (highest log2FC, S. Table 6) was observed for *NRXN1*, a gene coding for a membrane bounded cellular adhesion molecule [55], with reduced expression in schizophrenia MSCs cluster as compared to control MSCs (S. Fig. 2C).

The ependymal cluster exhibited the largest number of DEGs between schizophrenia and controls, including reduced *PDE4D*, and increased *FOXP2* and *EML6* expression activity in schizophrenia (S. Fig. 2A). The genes *PDE4D*, *FOXP2*, and *EML6* code for a cAMP phosphodiesterase [56], a transcription factor implicated in a speech and language disorder [57–59], and a microtubule associated protein [60, 61], respectively.

Pericytes were the second cluster with the highest number of DEGs between schizophrenia and controls (S. Fig. 2B), with increased *LRBA* and reduced *DOCK9* expression activity in schizophrenia pericytes. While *LRBA* codes for a lipopolysaccharide (LPS) responsive protein [62], *DOCK9* codes for a guanine nucleotide-exchange factor that enables cadherin binding activity and modulates filopodia formation and blood vessel morphogenesis [63].

The expression of *NRXN1*, *PDE4D*, *LRBA*, and *FOXP2* in the samples did not significantly correlate with any of the case-related variables (S. Table 7), suggesting that changes in the expression of these genes are mainly due to the effect of diagnosis.

Taken together, our results suggest that the relative abundance of the different main classes of BBB cell types is unaltered in the midbrain of the patients with schizophrenia, and that the few schizophrenia-associated changes in gene expression are in the ependymal, pericytes, and MSCs nuclei.

### Sub-clustering analysis of the major BBB cell types

To investigate if sub-populations of BBB cell types were altered in relation to schizophrenia, we ran sub-clustering analysis across all the BBB cell types containing more than 1000 nuclei and compared their proportions between schizophrenia and controls. Sub-clustering analysis of pericytes, fibroblasts, and ependymal nuclei did not reveal a schizophrenia-associated subpopulation (data not shown).

### Sub-clustering analysis revealed four different endothelial sub-populations

Midbrain endothelial sub-populations (Fig. 2A; 2244 endothelial nuclei) exhibited enriched expression of genes highly expressed by different human lung endothelial sub-populations [64] (Fig. 2B), indicating the presence of nuclei corresponding to arteries, capillaries, and two sub-types of veins in our dataset. Capillary nuclei comprised the largest midbrain endothelial population (56.7% ± 12.1; S. Table 11) and abundantly expressed *CLDN5* (Fig. 2C, S. Table 4). Arterial nuclei represented 20% ± 8.2 of the midbrain endothelial cells (S. Table 11) and highly expressed previously reported arterial markers, such as *VEGFC*, *EFNB2*, and *FBLN5* [65, 66], and novel markers, such as *IGFBP3, DKK2*, and *ROR1* (Fig. 2C, S. Table 4). Venular nuclei segregated into two sub-populations, both highly expressing the venular marker gene *TJP1*. Remarkably, Endo_veins1 (16.2% ± 7.3, S. Table 11) abundantly expressed the glutamate transporter *SLC1A1*, implying a possible role in the regulation of brain glutamate levels [67]. Endo_veins2 represented a smaller proportion of endothelial nuclei (7% ± 6.3, S. Table 11) and highly expressed *SERPINE1*, *VCAM1*, and *ICAM1* (Fig. 2C, S. Table 4), suggesting their involvement in the regulation of brain vascular capture and permeability to circulating immune cells [29, 68]. Accordingly, “Cell migration”, “Response to stimulus”, and “Signal transduction” were the top enriched biological processes in Endo_veins2 (Fig. 2D, S. Table 8).

To test for the presence of schizophrenia-related endothelial sub-populations, we compared the proportions of the endothelial sub-clusters between schizophrenia and controls, but no differences were observed (Fig. 2E).

In summary, these data provide a characterization of different endothelial sub-populations in the human midbrain, and none of them were differentially represented in the schizophrenia samples.

### Sub-clustering analysis revealed six astrocyte sub-populations

Variation in the sequence and expression activity of genes highly expressed by astrocytes has been associated with schizophrenia [69, 70]. We performed sub-clustering analysis of the astrocyte nuclei to describe the different sub-populations of astrocytes in the human midbrain, and to determine whether there is a contribution of a particular sub-population of astrocytes to schizophrenia pathophysiology.

**Fig. 3:**
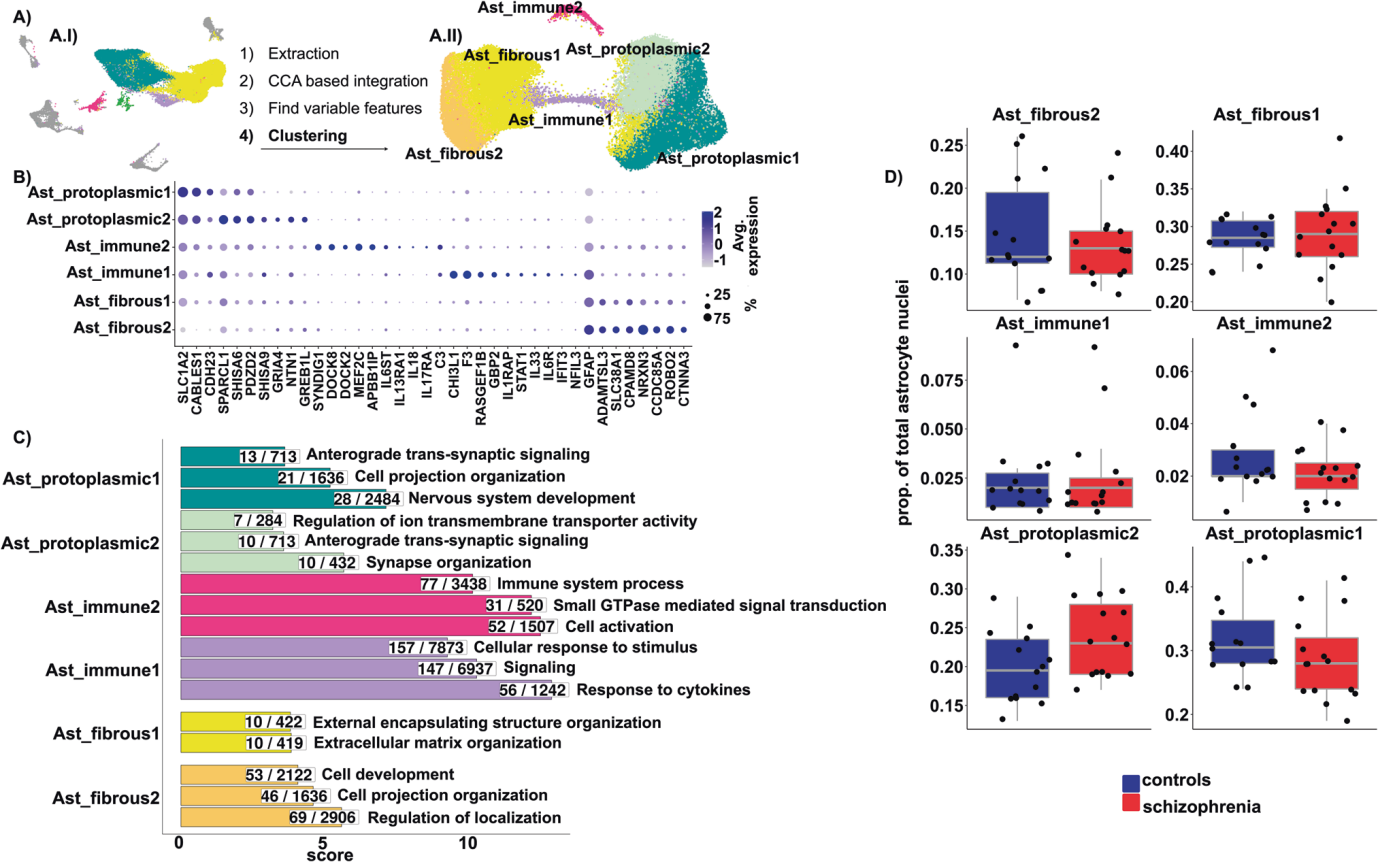
Identification and characterization of six astrocyte sub-populations. **A** Sub-clustering workflow. **A.I** UMAP highlighting astrocyte nuclei, which were extracted and used for sub-clustering analysis. **A.II** UMAP depicting 47,096 nuclei grouped in six astrocyte sub-clusters (dim = 20, k = 20, res = 0.2). **B** Dotplot depicting scaled average expression of representative marker genes of the identified astrocyte sub-populations. **C** Barplot depicting the top three most significantly enriched gene ontology terms, grouped by biological processes, in the more abundantly expressed genes (detailed in Methods) among the different astrocyte sub-populations. Redundancy of the terms was reduced by aggregating terms with high semantic similarity. **D** Boxplots depicting astrocyte sub-population proportions in schizophrenia and controls. Each dot indicates a sample and horizontal lines indicate the median. Group comparison was carried out with a generalized linear model and *p* values were Bonferroni corrected. No significant (adjusted *p* value < 0.05) differences were observed between schizophrenia and controls.

With low clustering resolution, the astrocyte nuclei segregated into six sub-clusters (Fig. 3A). Based on the expression of previously reported maker genes of astrocyte sub-types, such as *GFAP* for fibrous and *SLC1A2* for protoplasmic astrocytes [18, 71] (Fig. 3B, S. Table 5), and in gene ontology enrichment analysis (Fig. 3C, S. Table 9), we annotated the six sub-clusters into two protoplasmic (52.11%, S. Table 11), two fibrous (42.86%, S. Table 11), and two astrocyte sub-populations associated with immune functions (4.93%, S. Table 11). In Ast_protoplasmic2 compared to Ast_protoplasmic1, the genes *SHISA9*, *GRIA4*, *NTN1*, and *GREB1L* were more abundantly expressed (Fig. 3B). Ast_fibrous2 and Ast_fibrous1 nuclei were transcriptionally similar and shared several marker genes, like *GFAP*, *ADAMTSL3*, *SLC38A1*, *CPAMD8*, among others (Fig. 3B, S. Table 5); however, these genes were more abundantly expressed in Ast_fibrous2 (Fig. 3B).

The nuclei of the two immune-related astrocyte sub-clusters exhibited increased expression of the complement component 3 (*C3*) (Fig. 3B, S. Table 5). Ast_immune1 represented a small percentage of the astrocyte nuclei (2.5% ± 2.2, S. Table 11) and highly expressed genes coding for interferon-inducible proteins, such as *GBP2* [72], *IFIT3*, and *STAT1* (Fig. 3B, S. Table 5). We evaluated the expression of two lists of genes related to an astrocytic reactive phenotype, across the different astrocyte sub-types (S. Fig. 3). The first set corresponded to genes highly expressed by a sub-type of astrocytes identified in multiple sclerosis active lesions [73] (S. Fig. 3A.I–A.II), and the second set contained commonly up-regulated genes in human astrocytes subjected to different stressful stimuli [74] (S. Fig. 3B), resembling the transcriptome of a “general” astrocyte reactive phenotype. Ast_immune1 nuclei were enriched in both gene sets, as compared to the other midbrain astrocyte clusters, indicating that Ast_immune1 may correspond to reactive astrocytes that are present in low abundance in the human midbrain, normally and in schizophrenia. The second identified immune-related astrocyte sub-population, Ast_immune2, also represented a small proportion of the astrocytes (2.5% ± 1.4, S. Table 11) and highly expressed the genes coding for the Dedicator of Cytokinesis Proteins 2 and 8 (*DOCK2* and *DOCK8*; Fig. 3B, S. Table 5). These proteins are guanine nucleotide-exchange factors that activate Rho-family small GTPases on the plasma membrane of leukocytes and dendritic cells, modelling their migration [75]. Congruently, “Cell activation”, “Small GTPase mediated signal transduction”, and “Immune system process” were the top three enriched biological processes in Ast_immune2 (Fig. 3C, S. Table 9).

To evaluate whether a sub-population of midbrain astrocytes may be involved in schizophrenia, we compared the proportion of these sub-clusters between schizophrenia and controls. We did not observe changes in the proportion of any of the described astrocyte sub-population associated with schizophrenia (Fig. 3D), suggesting that the number of different astrocyte sub-types in the human midbrain is not altered in schizophrenia.

## Discussion

In the present study, we analyzed the BBB cells in the midbrain of patients with schizophrenia using snRNAseq, to investigate their possible contribution to schizophrenia brain pathology. Despite the identification of different sub-populations of pericytes, fibroblasts, ependymal, endothelial cells, and astrocytes, we found no change in their relative abundance associated with schizophrenia. This suggests that all the sub-types of cells contributing to the architecture of the BBB are present in the normative amounts in the midbrain of people with schizophrenia. Additionally, many of the cell types did not contain DEGs in the BBB of the schizophrenia midbrain as compared to controls. However, three cell types, the ependymal, pericytes, and MSCs, did contain some transcriptional changes.

Across the different BBB cell types, the largest difference in gene expression was detected for *NRXN1*, with reduced expression in schizophrenia MSCs cluster as compared to controls. Deletions in the coding region of *NRXN1* increase schizophrenia risk [76–78], which might be related to *NRXN1* expression activity [79]. NRXN1 is an adhesion molecule, mainly expressed in the presynaptic membrane of the neurons [55]. NRXN1 expression in the plasma membrane of non-neuronal cell types, such as astrocytes, has also been demonstrated [80, 81] and linked to the modulation of synaptic functioning through a neurexin-dependent mechanism distinct from the mechanisms of action of neural neurexins. Therefore, the reduced *NRXN1* expression in the MSCs cluster might suggest potential alterations in the regulation of synapse functioning by non-neuronal cells in schizophrenia. However, the MSCs cluster represented <1% of the BBB nuclei, thus it is difficult to estimate whether the reduction in *NRXN1* expression by MSCs would reflect a biologically meaningful difference.

Ependymal nuclei exhibited the largest number of DEGs, including reduced *PDE4D* and increased *FOXP2* expression in schizophrenia. Genetic variation in both genes, *FOXP2* and *PDE4D*, was previously associated with schizophrenia [82–85]. PDE4D is involved in cAMP degradation [56], a signal transduction molecule influencing a broad range of cellular functions [86, 87], whereas *FOXP2* encodes for the transcription factor forkhead box P2, which has been widely linked to speech and language development [57–59]. Verbal fluency deficits and disorganized speech are often observed in schizophrenia patients [88, 89], and may be related to altered *FOXP2* expression and/or functioning [90]. *FOXP2* expression was reduced in the prefrontal cortex of male schizophrenia patients [91], and we observed increased *FOXP2* expression in schizophrenia, particularly in the midbrain ependymal cells. These contrasting results might be explained due to cell type-specific effects of schizophrenia on *FOXP2* expression activity, which are only evidenced through single-cell analysis. Another transcript that schizophrenia ependymal nuclei had increased expression of was *EML6*. Genetic variation in *EML6* was associated with the density of calbindin-containing GABAergic neurons in the human prefrontal cortex [92], which are reduced in schizophrenia [93]. EML6 is a Microtubule Associated Protein [60, 61] potentially related to cilium organization and movement [94] and, while the role of midbrain ependymal cells in modulating the number of calbindin neurons in schizophrenia is not clear, the beating of ependymal cilia along the lateral ventricle is probably involved in neurogenesis [95]. In schizophrenia, it seems to be reduced neurogenesis capacity in the subependymal zone as compared to controls [33]. Therefore, the altered expression of *EML6* in the midbrain ependymal cells might reflect a general feature of schizophrenia ependymal cells, which could affect the beating of ependymal cilia and thus contribute to the reduced neurogenesis reported in the subependymal zone of schizophrenia patients.

Pericytes, which wrap around the capillaries, were the second cluster with the highest number of DEGs between schizophrenia and controls. In schizophrenia, the pericytes presented reduced expression of *DOCK9*. Besides the role of DOCK9 in promoting dendrite growth [96], it was implicated in the modulation of blood vessel morphology [63], and evidence derived from cortical and retinal vasculature indicate alterations in the morphology of the vessels and in the structure of the vascular network in schizophrenia [97–107]. The reduced expression of *DOCK9* in the pericytes of schizophrenia cases could affect the structure of the brain blood vessels, and this might not necessarily be reflected in the transcriptome of the vasculature. In contrast to reduced *DOCK9*, the expression of *LRBA* was higher in schizophrenia pericytes compared to controls. *LRBA* expression in B cells and macrophages is induced by bacterial LPSs and is relevant for vesicle trafficking and secretion [62]. Increased *LRBA* expression in schizophrenia derived pericytes might suggest an involvement of pericytes in the immune response to bacteria in the midbrain of schizophrenia patients [108]. Nevertheless, the total number of identified DEGs in the ependymal and pericytes was small to estimate potential alterations in their cellular functions associated with schizophrenia.

Some limitations in our experimental design might potentially explain the lack of widespread differences in the BBB between schizophrenia and controls. Possible transcriptional changes related to the identified DEGs in schizophrenia could be below our detection rate, limiting our ability to probe deeper into other associated transcripts and thus, limiting our ability to estimate potential alterations in the cellular functions associated with schizophrenia. In addition, transcriptomic analysis of brain samples from 559 schizophrenia patients revealed that changes in the expression of transcript splice variants captured the largest schizophrenia effect [109]. In our data, endothelial nuclei derived from schizophrenia exhibited reduced expression of *HNRNPA2B1*, a gene coding for a heterogeneous ribonucleoprotein involved in the alternative splicing of several genes [110–112] (S. Fig. 2D). However, as the 10X snRNAseq methodology does not allow for the detection of alternative spliced transcripts, our data do not contain alternative splicing information; thus, we cannot evaluate whether the reduced expression of *HNRNPA2B1* in schizophrenia affects the transcriptome of endothelial nuclei at the isoform-level.

In contrast to neurodegenerative diseases, such as Parkinson’s [113], Alzheimer’s [114], and frontotemporal dementia [115], where well-defined brain regions are more affected by the disease, the pathology of mental disorders like schizophrenia is more subtle and diffuse throughout many brain regions [116, 117]. This precludes a comparative analysis of affected and unaffected brain regions from the same donor. In addition, schizophrenia pathology may originate during early CNS development and in this study we analyzed adult *post-mortem* tissue; thus, some possible alterations in the brain vasculature and in the functioning of the BBB could have occurred in earlier stages of development [118] and may not be detectable in the adult brain. Also, there is considerable heterogeneity in the clinical features described among patients with schizophrenia [119–121], which may be reflected in gene expression activity [122], and that we are not addressing due to the lack of clinical information and a small sample size. Failure in properly accounting for clinical heterogeneity may compromise estimation of the schizophrenia effect on gene expression activity and thus, partially mask the differences between schizophrenia and controls.

To account for potential heterogeneity across the patients with schizophrenia, recent studies stratified the cohorts based on the inflammatory status of the patients and found that a large subgroup of schizophrenia patients exhibit increased expression of inflammatory cytokines in the brain (estimated ∼40%) [3, 123, 124]. Schizophrenia patients with a pro-inflammatory signature exhibited possible alterations in the brain vasculature and BBB permeability more consistently than the general schizophrenia population (discussed in [7]), reflected by altered expression of structural and functional brain endothelial cells marker genes, such as *ICAM1*, *CDH5*, *OCLN*, and *ABCG2* [4, 124]. Also, increased SERPINA3 expression in cortical astrocytes [125], and increased *GFAP* expression was reported in the midbrain of high-inflammation schizophrenia [5], whereas these differences were not observed between low-inflammation schizophrenia and control cases. Thus, it could be that major alterations in the brain endothelium and astrocytes occur in patients with an elevated pro-inflammatory profile and not in the general schizophrenia population. For the present study, we arbitrarily selected a subset of cases from the same cohort analyzed by Zhu et al. and Fillman et al. [3, 123], who described an increased pro-inflammatory status in almost half of the schizophrenia patients. In our study, only four of those high-inflammation schizophrenia cases were included by chance. Therefore, if major alterations in the BBB are presented exclusively during high-inflammation state, the reduced number of high-inflammation cases in our study might explain the limited differences observed between schizophrenia and control BBB cell types. Moreover, Harris et al. [35] investigated transcriptional changes in the brain vasculature using laser capture microscopy, also in a subset of cases from this same cohort as analyzed by Zhu et al. [3] and Fillman et al. [123]. After multiple comparisons correction, the authors reported no transcriptional differences between schizophrenia and control vasculature [35]. A potential small number of high-inflammation schizophrenia cases in the subset analyzed by Harris et al. [35] might also be an explanation for the lack of differences between schizophrenia and control brain vasculature reported by the authors.

In summary, our results suggest that the relative abundance of the BBB cell types and cellular sub-populations remains unaltered in schizophrenia pathology. Nonetheless, transcriptional changes in the midbrain BBB cells associated with schizophrenia are found, but are limited and are specific to two cell types, the ependymal and pericytes. A future single-cell transcriptomic study of the BBB in schizophrenia patients with an elevated pro-inflammatory status could help to identify the BBB cell types contributing to schizophrenia brain pathology in the high-inflammation subgroup of patients, which represents an important proportion of the schizophrenia population.

## Supplementary information

Supplementary information and figures

Supplementary Table 1 and 2

Supplementary Table 3

Supplementary Table 4

Supplementary Table 5

Supplementary Table 6

Supplementary Table 7

Supplementary Table 8

Supplementary Table 9

Supplementary Table 10 and 11

## Data Availability

All the data are available through the SMRI website, www.stanleyresearch.org or directly at www.sncid.stanleyreserach.org.
