# Supplementary material for: Single-nucleus RNA sequencing of midbrain blood-brain barrier cells in schizophrenia reveals subtle transcriptional changes with overall preservation of cellular proportions and phenotypes
Source: Mol Psychiatry. 2022 Oct 3;27(11):4731–40. doi: 10.1038/s41380-022-01796-0 (PMC9734060; doi:10.1038/s41380-022-01796-0)
Supplement: Supplementary file 1 — Supplementary information and figures [file 41380_2022_1796_MOESM1_ESM.docx]

**Supplementary information**

Supplementary figures





**Supplementary Figure 1. Identification of the major brain cell types.**

1. UMAP depicting 178 009 nuclei. Colors indicate the different major brain cell types.
2. Dotplot depicting scaled average expression of representative marker genes of the major brain cell types.
3. Violin with boxplots depicting module scores for gene sets associated with mouse astrocytes and ependymal cells [1], in our human midbrain astrocyte and ependymal clusters.

**Supplementary Figure 2. Differentially expressed genes between schizophrenia and controls across the major BBB cell types.**





A-D) Violin with boxplots depicting expression levels (log normalized counts) of the identified differentially expressed genes (absolute log_2_FC > 0.3 and FDR adjusted *p* value < 0.05, detailed in Methods) between schizophrenia and controls, across the different major BBB cell types. The arrows indicate the direction of change in gene expression activity in schizophrenia with respect to controls.

**Supplementary Figure 3. Gene sets related to human reactive astrocytes are enriched in Ast_immune1 sub-population.**





1. Comparison of Ast_immune1 with a human reactive astrocyte sub-type enriched in active multiple sclerosis (MS) lesions (cluster1 in Absinta et al., (2021) [2]). A.I) Top: Violin with boxplots depicting module scores for a gene set associated with a human reactive astrocyte sub-type, in our human midbrain astrocyte sub-populations. Bottom: UMAP plots depicting module score in each nucleus. A.II) Venn diagram depicting the number of shared genes between the top 100 marker genes of Ast_immune1 (based on fold change) and the top 100 marker genes of the human reactive astrocyte sub-type [2].
2. Ast_immune1 is enriched in a gene set derived from a meta-analysis of genome-wide expression studies of astrocytes subjected to diverse stressful conditions [3]. Top: Violin plots with boxplots depicting module scores for the set of commonly up-regulated genes in human astrocytes under stressful stimuli. Bottom: UMAP plots depicting module score in each nucleus.

References

1. Zywitza, V., A. Misios, L. Bunatyan*, et al.*, *Single-Cell Transcriptomics Characterizes Cell Types in the Subventricular Zone and Uncovers Molecular Defects Impairing Adult Neurogenesis.* Cell Rep, 2018. **25**(9): p. 2457-2469.e8.

2. Absinta, M., D. Maric, M. Gharagozloo*, et al.*, *A lymphocyte–microglia–astrocyte axis in chronic active multiple sclerosis.* Nature, 2021. **597**(7878): p. 709-714.

3. González-Giraldo, Y., D.A. Forero, G.E. Barreto*, et al.*, *Common genes and pathways involved in the response to stressful stimuli by astrocytes: A meta-analysis of genome-wide expression studies.* Genomics, 2021. **113**(2): p. 669-680.

Supplementary table titles

**Supplementary Table 1.** Information about the cases used in this study.

**Supplementary Table 2.** Comparison of case-related variables between schizophrenia and controls.

**Supplementary Table 3.** Excel table indicating all highly expressed genes per BBB cell type.

**Supplementary Table 4.** Excel table indicating all highly expressed genes per endothelial sub-population.

**Supplementary Table 5.** Excel table indicating all highly expressed genes per astrocyte sub-population.

**Supplementary Table 6.** Excel table indicating all differentially expressed genes in schizophrenia as compared to controls in each BBB cell type.

**Supplementary Table 7.** Correlations between gene expression levels and case-related variables.

**Supplementary Table 8.** Excel table with significantly enriched gene ontology terms per endothelial sub-population.

**Supplementary Table 9.** Excel table with significantly enriched gene ontology terms per astrocyte sub-population.

**Supplementary Table 10.** Relative abundance of the major brain cell types in the snRNAseq dataset.

**Supplementary Table 11.** Relative abundance of the major BBB cell types, endothelial and astrocyte sub-populations in the human midbrain.
