# Supplementary material for: Single-nucleus RNA sequencing of midbrain blood-brain barrier cells in schizophrenia reveals subtle transcriptional changes with overall preservation of cellular proportions and phenotypes
Source: Mol Psychiatry. 2022 Oct 3;27(11):4731–40. doi: 10.1038/s41380-022-01796-0 (PMC9734060; doi:10.1038/s41380-022-01796-0)
Supplement: Supplementary file 2 — Supplementary Table 1 and 2 [file 41380_2022_1796_MOESM2_ESM.docx]

**Supplementary Table 1.** Information about the cases used in this study.

| **Code** | **Diagnose** | **Age of death** | **Sex** | **Cause of death** | **Age of onset** | **Lifetime AP (mg)** | **PMI** | **BrainPH** | **RIN** | **NeuN+ nuclei** | **DN nuclei** | **Seq. saturation** | **% Transcripts mapped to the genome** |
| --- | --- | --- | --- | --- | --- | --- | --- | --- | --- | --- | --- | --- | --- |
| 1 | CN | 30-40 | 1 | CARDIAC | NA | NA | 22 | 6.48 | 5.6 | 5 000 | 40 000 | 70.2 | 58.7 |
| 2 | CN | 30-40 | 2 | CARDIAC | NA | NA | 24 | 6.87 | 7.9 | 1 775 | 40 000 | 41.3 | 62.2 |
| 3 | CN | 41-51 | 1 | CARDIAC | NA | NA | 29 | 6.94 | 6.7 | 5 000 | 40 000 | 65 | 55.5 |
| 4 | CN | 41-51 | 1 | CARDIAC | NA | NA | 21 | 6.81 | 6.6 | 5 000 | 40 000 | 56.4 | 48.7 |
| 5 | CN | 30-40 | 1 | MYOCARDITIS | NA | NA | 52 | 6.7 | 7.7 | 5 110 | 40 000 | 69.8 | 54.2 |
| 6 | CN | 52-62 | 1 | CARDIAC | NA | NA | 47 | 6.8 | 7.3 | 5 000 | 40 000 | 44.5 | 42.2 |
| 7 | CN | 30-40 | 2 | CARDIAC | NA | NA | 28 | 6.7 | 7.5 | 5 000 | 40 000 | 71.3 | 53.4 |
| 8 | CN | 30-40 | 2 | CARDIAC | NA | NA | 33 | 6 | 7.7 | 8 000 | 39 000 | 66 | 53.1 |
| 9 | CN | 52-62 | 1 | CARDIAC | NA | NA | 28 | 6 | 7.1 | 3 500 | 40 000 | 67.7 | 36.1 |
| 10 | CN | 41-51 | 1 | CARDIAC | NA | NA | 31 | 6.7 | 8.6 | 5 000 | 40 000 | 38.6 | 40.9 |
| 11 | CN | 30-40 | 1 | CARDIAC | NA | NA | 13 | 6.5 | 4.1 | 5 000 | 40 000 | 35 | 38.6 |
| 12 | CN | 52-62 | 1 | CARDIAC | NA | NA | 9 | 6.4 | 8.6 | 9 490 | 40 000 | 45.2 | 30.4 |
| 13 | CN | 41-51 | 1 | CARDIAC | NA | NA | 46 | 6.5 | 8.5 | 10 000 | 40 000 | 42.5 | 48.8 |
| 14 | CN | 41-51 | 2 | CARDIAC | NA | NA | 28 | 6.59 | 7.4 | 10 000 | 40 000 | 41.8 | 42 |
| 15 | SZ | 41-51 | 1 | PNEUMONIA | 21 | 180 000 | 26 | 6.42 | 5.3 | 5 000 | 40 000 | 67.4 | 58.6 |
| 16 | SZ | 41-51 | 2 | SUIC:JUMPED | 34 | 20 000 | 52 | 6.51 | 8.9 | 10 000 | 40 000 | 76.1 | 75 |
| 17 | SZ | 30-40 | 1 | CARDIAC | 19 | 20 000 | 29 | 6.5 | 7.5 | 5 000 | 40 000 | 62.8 | 59.4 |
| 18 | SZ | 30-40 | 1 | MVA | 17 | 120 000 | 80 | 6.6 | 6.9 | 5 000 | 41 600 | 37.2 | 35.7 |
| 19 | SZ | 30-40 | 1 | CARDIAC | 13 | 20 000 | 30 | 6.8 | 8.1 | 5 000 | 40 000 | 61.7 | 54.4 |
| 20 | SZ | 41-51 | 1 | CIRRHOSIS | 18 | 90 000 | 18 | 6.3 | 6.9 | 10 000 | 38 000 | 59.6 | 56.1 |
| 21 | SZ | 19-29 | 1 | OD | 18 | 2 500 | 28 | 6.73 | 8.4 | 10 000 | 35 000 | 64.7 | 60.5 |
| 22 | SZ | 41-51 | 1 | CARDIAC | 31 | 34 000 | 9 | 6.2 | 5.1 | 5 000 | 40 000 | 59.5 | 41.5 |
| 23 | SZ | 19-29 | 1 | SUIC:OD | 20 | 12 000 | 15 | 6.2 | 8.5 | 5 000 | 40 000 | 67.8 | 51 |
| 24 | SZ | 52-62 | 1 | CARDIAC | 28 | 100 000 | 10 | 6.1 | 7.7 | 2 570 | 42 560 | 58 | 35.2 |
| 25 | SZ | 52-62 | 2 | CARDIAC | 29 | 15 000 | 13 | 6.49 | 8.9 | 10 000 | 35 000 | 56.1 | 55.2 |
| 26 | SZ | 41-51 | 1 | CARDIAC | 23 | 130 000 | 43 | 6.63 | 7.5 | 10 000 | 40 000 | 45.1 | 54.7 |
| 27 | SZ | 30-40 | 1 | PNEUMONIA | 21 | 75 000 | 34 | 6.18 | 8.8 | 9 040 | 40 000 | 77.4 | 56.8 |
| 28 | SZ | 30-40 | 1 | CARDIAC | 22 | 35 000 | 33 | 6.2 | 8.1 | 5 000 | 40 000 | 44.9 | 32.5 |
| 29 | SZ | 41-51 | 1 | EXHAUSTIVE MANIA/NMS | 19 | 350 000 | 9 | 5.9 | 7.2 | 10 430 | 28 530 | 66.7 | 35.1 |

Information about the cases and quality measurements of the midbrain samples obtained from them. Schizophrenia (SZ), Control (CN), Antipsychotics (AP), Post Mortem Interval (PMI), Double Negative (DN), Sequencing (Seq), Over Dose (OD). Lifetime AP: fluphenazine equivalents.

**Supplementary Table 2.** Comparison of case-related variables between schizophrenia and controls.

| **Variable** | **Normality** | **Hom_var** | **Test** | ***p* value** |
| --- | --- | --- | --- | --- |
| Age | TRUE | TRUE | t-test | 0.27 |
| PMI | FALSE | TRUE | Man-Whitney U | 0.73 |
| BrainPH | TRUE | TRUE | t-test | 0.08 |
| RIN | FALSE | TRUE | Man-Whitney U | 0.38 |
| Sex | NA | NA | Fisher exact | 0.39 |

Results of comparing case-related variables between schizophrenia and controls. Homoscedasticity of the variance (Hom_var).
