# Supplementary material for: Single-nucleus RNA sequencing of midbrain blood-brain barrier cells in schizophrenia reveals subtle transcriptional changes with overall preservation of cellular proportions and phenotypes
Source: Mol Psychiatry. 2022 Oct 3;27(11):4731–40. doi: 10.1038/s41380-022-01796-0 (PMC9734060; doi:10.1038/s41380-022-01796-0)
Supplement: Supplementary file 7 — Supplementary Table 7 [file 41380_2022_1796_MOESM7_ESM.docx]

**Supplementary Table 7.** Correlations between gene expression levels and case-related variables.

| **Gene** | **Age** | ***p* value** | **pH** | ***p* value** | **AP** | ***p* value** | **PMI** | ***p* value** | **RIN** | ***p* value** | **sex** | ***p* value** |
| --- | --- | --- | --- | --- | --- | --- | --- | --- | --- | --- | --- | --- |
| AC013470.2 | -0.02 | 0.93 | 0.08 | 0.7 | -0.09 | 0.75 | 0.16 | 0.4 | 0.39 | ***0.04** | 0.18 | 0.35 |
| DOCK9 | 0 | 0.99 | 0.02 | 0.92 | -0.02 | 0.94 | 0.17 | 0.38 | 0.49 | ***0.01** | 0.2 | 0.29 |
| EML6 | 0.24 | 0.21 | -0.31 | 0.1 | 0.11 | 0.68 | -0.08 | 0.7 | -0.25 | 0.18 | -0.41 | ***0.03** |
| FOXP2 | 0.05 | 0.8 | 0.16 | 0.4 | -0.15 | 0.58 | 0 | 1 | 0.1 | 0.6 | 0.1 | 0.6 |
| HNRNPA2B1 | 0 | 1 | 0.48 | ***0.01** | -0.13 | 0.65 | -0.03 | 0.88 | 0 | 0.98 | 0.3 | 0.11 |
| ITGA1 | 0.07 | 0.73 | -0.2 | 0.31 | 0.2 | 0.47 | 0.01 | 0.95 | 0.26 | 0.17 | 0.1 | 0.6 |
| KCND2 | 0.32 | 0.09 | 0.04 | 0.85 | -0.21 | 0.45 | -0.16 | 0.4 | -0.32 | 0.09 | -0.05 | 0.8 |
| LMNTD1 | 0.13 | 0.52 | -0.08 | 0.7 | -0.06 | 0.83 | 0.2 | 0.29 | 0.41 | ***0.03** | 0.44 | ***0.02** |
| LRBA | 0.23 | 0.24 | -0.13 | 0.51 | 0.18 | 0.51 | -0.11 | 0.57 | 0.01 | 0.95 | -0.05 | 0.81 |
| NEK10 | -0.16 | 0.41 | 0.27 | 0.16 | -0.08 | 0.79 | 0.32 | 0.09 | 0.2 | 0.31 | 0.21 | 0.26 |
| NRXN1 | 0.37 | 0.05 | -0.07 | 0.72 | -0.09 | 0.74 | -0.33 | 0.08 | -0.29 | 0.12 | -0.23 | 0.22 |
| PDE4D | 0.18 | 0.36 | -0.21 | 0.27 | -0.2 | 0.47 | 0.04 | 0.82 | 0.04 | 0.83 | 0.15 | 0.44 |
| RUNX1T1 | 0.31 | 0.1 | -0.09 | 0.65 | 0.05 | 0.87 | -0.2 | 0.3 | 0.09 | 0.66 | -0.15 | 0.44 |
| TXNIP | 0.05 | 0.78 | -0.28 | 0.13 | 0.13 | 0.64 | -0.23 | 0.24 | 0.27 | 0.16 | 0.01 | 0.97 |

Results of correlating the average expression per sample of the identified differentially expressed genes between schizophrenia and controls (indicated in S. Figure 2), with the different case-related variables. A Pearson or Spearman correlation was performed, depending on whether the case-related variable was normally distributed. Significant correlations (*p* value < 0.05) are highlighted in bold. Antipsychotics (AP).
