# Supplementary material for: Single-nucleus RNA sequencing of midbrain blood-brain barrier cells in schizophrenia reveals subtle transcriptional changes with overall preservation of cellular proportions and phenotypes
Source: Mol Psychiatry. 2022 Oct 3;27(11):4731–40. doi: 10.1038/s41380-022-01796-0 (PMC9734060; doi:10.1038/s41380-022-01796-0)
Supplement: Supplementary file 10 — Supplementary Table 10 and 11 [file 41380_2022_1796_MOESM10_ESM.docx]

**Supplementary Table 10.** Relative abundance of the major brain cell types in the snRNAseq dataset.

| **Cell type** | **% of the major brain cell types** | **sd** |
| --- | --- | --- |
| Astrocytes | 36.34 | 14.56 |
| Endothelial | 1.34 | 0.97 |
| Fibroblasts | 2.48 | 2.31 |
| Pericytes-SMCs | 1.1 | 0.67 |
| Ependymal | 0.97 | 0.63 |
| Lymphocytes | 4.07 | 3.35 |
| Microglia | 20.86 | 8.64 |
| CAMs | 1.14 | 1.03 |
| Neurons | 22.93 | 9.44 |
| OPCs | 0.83 | 1 |
| Oligodendrocytes | 7.9 | 5.39 |

Mean proportions of the different major brain cell types, across the 29 cases. Standard deviation (sd).

**Supplementary Table 11.** Relative abundance of the major BBB cell types, endothelial and astrocyte sub-populations in the human midbrain.

| **Cell type** | **% of total BBB nuclei** | **sd** |
| --- | --- | --- |
| Astrocytes | 81.07 | 13.47 |
| Endothelial | 4.41 | 3.63 |
| Fibroblasts | 6.90 | 5.87 |
| Ependymal | 3.00 | 2.00 |
| Pericytes | 2.72 | 1.73 |
| SMCs | 1.07 | 0.84 |
| MSCs | 0.79 | 2.48 |
| **Sub-population** | **% of total endothelial nuclei** | **sd** |
| Endo_veins2 | 6.97 | 6.29 |
| Endo_veins1 | 16.21 | 7.25 |
| Endo_capillaries | 56.69 | 12.10 |
| Endo_arteries | 20.03 | 8.19 |
| **Sub-population** | **% of total astrocyte nuclei** | **sd** |
| Ast_fibrous2 | 14.03 | 5.35 |
| Ast_fibrous1 | 28.83 | 4.18 |
| Ast_immune1 | 2.45 | 2.21 |
| Ast_immune2 | 2.48 | 1.38 |
| Ast_protoplasmic2 | 21.90 | 5.26 |
| Ast_protoplasmic1 | 30.21 | 6.63 |

Mean proportions of the different major BBB cell types, endothelial and astrocyte sub-populations, across the 29 cases. Standard deviation (sd).
